# Spinal Cord Stimulation for Prolonged Disorders of Consciousness: A Study on Scalp Electroencephalography

**DOI:** 10.1111/cns.70180

**Published:** 2024-12-30

**Authors:** Jian Sun, Jiuqi Yan, Liang Zhao, Xiang Wei, Chang Qiu, Wenwen Dong, Bei Luo, Wenbin Zhang

**Affiliations:** ^1^ Department of Functional Neurosurgery The Affiliated Brain Hospital of Nanjing Medical University Nanjing China

**Keywords:** ABCD model, CRS‐R, disorders of consciousness, EEG power spectrum, Event‐related potentials, P300, spinal cord stimulation

## Abstract

**Background:**

Patients with disorders of consciousness (DOC) undergoing spinal cord stimulation (SCS) for arousal treatment require an assessment of their conscious state before and after the procedure. This is typically evaluated using behavioral scales (CRS‐R), but this method can be influenced by the subjectivity of the physician. Event‐related potentials (ERP) and EEG power spectrum are associated with the recovery of consciousness. This study aims to explore the electrophysiological and behavioral evidence of consciousness recovery in DOC patients after spinal cord stimulation (SCS) and to investigate the role of scalp EEG as a guide for preoperative assessment related to the surgery.

**Methods:**

For the 27 recruited patients, the CRS‐R scale assessment and ERP P300 evaluation were completed before the surgery. At 3 months post‐surgery, all 27 patients underwent the same assessments as preoperatively, and at 6 months post‐surgery, the same evaluations were repeated for the 15 patients who could still be followed up. Between May 2023 and November 2023, resting‐state EEG was collected from 13 patients using a 19‐channel setup, with additional resting‐state EEG recordings taken at 3 months and 6 months after the surgery. The EEG data were processed using EEGLAB to obtain P300‐related metrics and EEG power spectrum. Changes in the CRS‐R scale, ERP, and EEG power spectrum before and after the surgery were compared.

**Results:**

The Behavioral Scale (CRS‐R) showed significant improvement at 3 months and 6 months post‐surgery compared to preoperative assessments, with statistical significance (*p* < 0.001). The resting‐state EEG power in the 5–9 Hz frequency band demonstrated statistically significant improvements at the P3 and O1 electrodes; however, this statistical result do not survive FDR correction. In the 9–13 Hz and 20–35 Hz frequency bands, the power spectrum showed statistically significant improvements across most electrodes of the brain, and these results survive FDR correction (*p* < 0.05). The mean amplitude, peak, and latency of P300 at the Pz electrode showed significant improvements at 3 months and 6 months post‐surgery compared to preoperative values, with statistical significance (*p* < 0.05).

**Conclusion:**

Our study shows that SCS can effectively improve the consciousness states of patients with DOC. After surgery, there were positive changes in the EEG power spectrum of the patients, transitioning from type “B” to better types “C” and “D.” The average amplitude, peak, and latency of P300 also demonstrated significant improvements postoperatively. We believe that the “ABCD” model and ERP assessment applied during the preoperative evaluation can effectively enhance the success rate of SCS surgery in promoting awakening.

## Graphical abstract

Our study indicates that spinal cord stimulation improves consciousness in DOC patients, shown by EEG changes and enhanced P300 metrics. The “ABCD” model and ERP assessments may boost SCS surgery success rates for awakening.

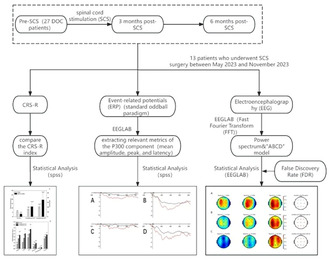

## 1 Introduction

DoC stands for a class of severe neurological conditions that can essentially be categorized into coma, vegetative state (VS), also termed unresponsive wakefulness syndrome (UWS), or minimally conscious state (MCS) [1]. The term VS/UWS denotes a condition of wakefulness without (clinical signs of) awareness [2]. Such patients may open their eyes but exhibit only reflex (i.e., non‐intentional) behaviors and are therefore considered unaware of themselves and their surroundings. Patients in MCS usually show more consciousness‐related behaviors than those in VS/UWS, such as visual pursuit, localization of noxious stimulation, simple emotional responses to external stimuli and command‐following behaviors [3]. The differentiation between VS/UWS and MCS is most probably gradual (continuous) rather than binary (all‐or‐none) [4], and some survivors with VS/UWS may recover to MCS or better, even years after the brain injury [5, 6, 7, 8]. The heterogeneity of the MCS is now recognized, and consequently patients may be classified according to the degree of their behavioral responses into MCS plus (i.e., if they are able to follow commands, produce intelligible words and/or display intentional communication) or minus (e.g., if they only show voluntary signs of consciousness such as localization to pain or visual pursuit but no behaviors suggestive of language processing) [9]. Patients who recover functional communication or functional object use are considered as ‘emerged from MCS’ [10].

Treatment of DoC remains a great challenge confronting patients and doctors, and multiple therapeutic approaches have been proposed in recent years, including pharmacological treatments (e.g., amantadine, zolpidem, and baclofen), transcranial direct current stimulation, and invasive neuromodulation [11]. The spinal cord stimulation (SCS) as an effective neuromodulation technology has attracted a growing attention in DOC field. Spinal cord stimulation(SCS) is a valuable technique for the rehabilitation of DOC patients because it is a simpler, less‐invasive surgical procedure than deep brain stimulation(DBS) [12]. For SCS treatment, doctors implant electrodes to patients' epidural space of the cervical vertebrae at C2—C4 levels by neurosurgical procedure. The electrical pulses released by the electrodes modulate nerve activity. SCS has achieved outstanding results in the treatment of pain. Compared to non‐invasive methods, SCS can directly stimulate central nervous system to provide more powerful stimulation effect [13]. Meanwhile, SCS is a feasible invasive method because of low cost, low risk, and easy postoperative management [14].

Currently, the most widely accepted tool for assessing consciousness is a behavioral scale. The Coma Recovery Scale‐Revised (CRS‐R) is a standardized assessment tool used to measure the level of consciousness in comatose patients [15]. After its revision in 2004, most of the articles used CRS‐R, a 15‐item scale that evaluates the patient's responses to auditory, visual, motor, and verbal stimuli. It is a reliable and valid measure of consciousness [16] that can be used to assess the level of consciousness and predict patient outcomes accurately. The CRS‐R can also be used to monitor the progress of patients with disorders of consciousness as they undergo treatment. Additionally, some studies have proposed the use of the CRS‐R index [17] to assess the level of consciousness in patients with disorders of consciousness, finding that the CRS‐R index can effectively differentiate between Unresponsive Wakefulness Syndrome (UWS) and Minimally Conscious State (MCS), making it an effective assessment tool.

Recently, event‐related potential (ERP) recording has been used as an objective and easy evaluation method for assessing cortical information processing capabilities in the absence of overt behavior in patients with DOCs. Some studies have attempted to specifically detect the existence of attentional capabilities by measuring the P300 wave, which is well understood to be a correlate of attention and conscious perception [18, 19].

Some studies have indicated, an educated inspection of basic EEG features in prolonged DoC can provide relevant information about the functional integrity of thalamocortical networks [20]. One such hypothesis, the mesocircuit model, theorizes that the globus pallidus interna (GPi) is disinhibited following diffuse brain injury, and thus, silences the central thalamus, resulting in functional deafferentation of the cortex [21, 22]. Thus, recovery from DOC requires restoration of striatal functioning and thalamocortical integrity. The latter may be inferred from EEG, given that cortical oscillations such as theta and alpha are thought to be driven by the thalamus [23, 24, 25, 26, 27, 28]. As such, the loss and recovery of thalamocortical integrity is visible in noninvasive recordings and motivates the “ABCD” model by Schiff. The proposed ‘ABCD’ model [20, 29] shows the correlation of coarsely divided levels of cerebral deafferentation in severe brain injuries and the EEG power spectrum changes predicted to occur based on functional changes within neocortical and thalamic neurons as a result of the deafferentation patterns. Hence, depending on the degree of deafferentation, neurons of the central thalamus can be either quiescent (“A‐type”, “B‐type”), enter in a bursting mode (“C‐type”) or progress towards a tonic mode of activity (“D‐type”). Physiological correlates, in the form of shifts from B‐type to C‐type or D‐type dynamics, associated with the transition from VS/UWS to MCS and higher levels of recovery, have been seen in some medication‐responsive patients [30] and in patients who show spontaneous recovery during the acute [31, 32] or subacute‐to‐chronic [33] stage of DoC. Recovery from VS/UWS or MCS to a confusional state or higher levels of cognitive function is typically associated with restoration of D‐type dynamics [34, 35].

Given the importance of ERP and EEG power spectrum in assessing electrophysiological changes related to consciousness recovery, we aim to explore the electrophysiological and behavioral evidence of consciousness recovery in DOC patients after spinal cord stimulation (SCS).

## 2 Materials and Methods

### 2.1 Patients

In this study, we recruited 27 DOC patients, aged between 22 and 68 years (mean age = 49.5, SD = 13.2), The etiologies included hemorrhage in 15 cases, hypoxia in 2 cases, and trauma in 10 cases. The time from onset to surgery ranged from 2 to 13 months (mean duration = 5.0, SD = 3.2). All patients were clinically stable and were not receiving other treatments or medications that could alter cortical excitability. The clinical characteristics of the patients are presented in Table 1. Written informed consent for participation in the study was obtained from the patients' families. The study has been approved by the Ethics Committee of Nanjing Brain Hospital.

**TABLE 1:** Description of general conditions of the included DOCs.

Patients	Gender	Age	Months	Etiology
1	M	22	12	Traumatic
2	M	36	4	Hemorrhagic
3^a^	M	35	3	Hemorrhagic
4	M	62	3	Hemorrhagic
5^a^	F	23	4	Traumatic
6	M	57	2	Hemorrhagic
7	M	48	4	Anoxic
8	F	59	3	Traumatic
9^a^	M	40	13	Hemorrhagic
10	F	59	13	Hemorrhagic
11	M	33	4	Traumatic
12	M	37	7	Hemorrhagic
13	F	59	4	Hemorrhagic
14	M	68	7	Hemorrhagic
15^a^, ^b^	F	51	4	Traumatic
16^a^, ^b^	F	55	5	Anoxic
17^a^, ^b^	M	58	7	Traumatic
18^a^, ^b^	F	65	2	Hemorrhagic
19^a^, ^b^	M	53	2	Hemorrhagic
20^a^, ^b^	F	64	2	Traumatic
21^b^	M	47	7	Hemorrhagic
22^a^, ^b^	F	66	4	Traumatic
23^a^, ^b^	F	60	2	Hemorrhagic
24^a^, ^b^	M	50	4	Traumatic
25^a^, ^b^	F	47	4	Hemorrhagic
26^a^, ^b^	M	52	7	Hemorrhagic
27^a^, ^b^	M	31	3	Traumatic

^a^ Patients who completed the second ERP assessment 6 months after surgery.

^b^ Patients who completed the “ABCD” model assessment before surgery.

### 2.2 Behavioral Assessment

After admission, the patients with DOCs were allowed to familiarize themselves with the ward environment for 1–2 days. Once the patients' clinical conditions stabilized, a trained and experienced physician performed assessments using the Coma Recovery Scale‐Revised (CRS‐R). The CRS‐R is a sensitive tool for describing the level of consciousness in DoC patients and monitoring neurobehavioral recovery. In our study, each patient was assessed by the same physician both before and after the spinal cord stimulation (SCS) surgery. To ensure the patients were in an optimal state of alertness, clinical evaluations were conducted in the morning at the bedside following routine care procedures.

### 2.3 Electrophysiological Assessment

#### 2.3.1 Electroencephalography

A 19‐channel electrode cap (Neuroscan Inc., Charlotte, North Carolina, USA) was used according to the international 10–20 system. Reference electrodes were placed on both earlobes, and a ground electrode was placed at the center of the forehead. Throughout the experiment, electrode impedance was maintained below 40 kΩ. The bandpass filter was set from 0.1 to 100 Hz, with a sampling rate of 500 Hz. A 3‐min resting‐state electroencephalogram (EEG) was recorded while the patient was awake with eyes open. Prior to the assessment, the patient's bilateral trapezius muscles were gently stimulated to ensure they were alert. The assessment equipment used was the Nvx52 EEG amplifier (Nanjing Left and Right Brain Medical Technology Group Co. Ltd).

#### 2.3.2 Power Spectrum Analysis

The relevant analysis was conducted using the EEGLAB toolbox in MATLAB. First, the collected EEG data were preprocessed by applying filters to extract the required frequency bands for this study (high‐pass filter set at 1 Hz and low‐pass filter set at 50 Hz). The EEG data were then segmented into epochs, each lasting 2 s. Independent Component Analysis (ICA) was applied to remove artifact components from the EEG signals, and manually, some segments with significant interference were discarded. Subsequently, Fast Fourier Transform (FFT) was used in EEGLAB to obtain the power spectral density of the EEG. Paired sample *t*‐tests were performed on the power spectra before and after surgery, and the statistical results were corrected using the False Discovery Rate (FDR) method to derive the relevant outcomes.

#### 2.3.3 ERP Assessment

We used a standard oddball paradigm [36], employing an 800 Hz tone as the standard stimulus and a 1000 Hz tone as the deviant stimulus. The tones lasted for 100 ms, and patients were informed that they would hear a series of sounds and only needed to listen. Stimuli were presented in a random order with an inter‐stimulus interval of 0.8 to 1.2 s. Each block consisted of 200 stimuli, with the probability of the deviant stimulus being 0.2. The presentation order of the two paradigms was balanced across all patients. The two paradigms lasted approximately 6 min in total. Recordings were made from midline electrodes Fz, Cz, and Pz using a 19‐channel electrode cap (Neuroscan Inc., Charlotte, North Carolina, USA) according to the international 10–20 system. Reference electrodes were placed on both earlobes, and a ground electrode was placed at the center of the forehead. Throughout the experiment, electrode impedance was maintained below 30 kΩ. The bandpass filter was set from 0.1 to 100 Hz with a sampling rate of 2000 Hz. The assessment was conducted while the patient was awake, with eyes open, in a minimally noisy environment.

#### 2.3.4 ERP Analysis

Similarly, preprocessing was performed using the EEGLAB toolbox in MATLAB; however, in this case, the high‐pass filter was set to 0.1 Hz and the low‐pass filter was set to 30 Hz. Using ERPLAB, each EEG was divided into segments ranging from 200 ms before the stimulus to 1000 ms after the stimulus. The averaging calculations were then conducted in ERPLAB, extracting relevant metrics of the P300 component, such as mean amplitude, peak, and latency from the Fz, Cz, and Pz electrodes. These metrics were subjected to statistical calculations using SPSS software.

#### 2.3.5 Statistical Analysis

Statistical analyses were conducted using SPSS 25.0 on the behavioral test scores (CRS‐R) and ERP data (mean amplitude, peak, and latency of P300). Due to the small sample size in this study, the Shapiro–Wilk test was used to verify the normality of the data distribution. For normally distributed data, the results are expressed as mean ± standard deviation, and paired sample t‐tests were performed for pre‐ and post‐operative comparisons. For skewed data, results are presented as median (first quartile, third quartile) [M (Q1, Q3)], and the Wilcoxon signed‐ranks test was used to analyze pre‐ and post‐operative differences. A significance level of *p* < 0.05 was considered statistically significant.

## 3 Results

### 3.1 Patients' Characteristics

Only one patient experienced skin breakdown at the site of the chest wall battery pouch due to poor nutrition after surgery. Following a second surgery to move the battery to the left axilla, the patient's chest wall skin gradually healed, and no similar issues occurred thereafter. In all other patients, no adverse effects potentially related to SCS were observed.

### 3.2 CRS‐R and CRS‐R Index Results

**FIGURE 1:**
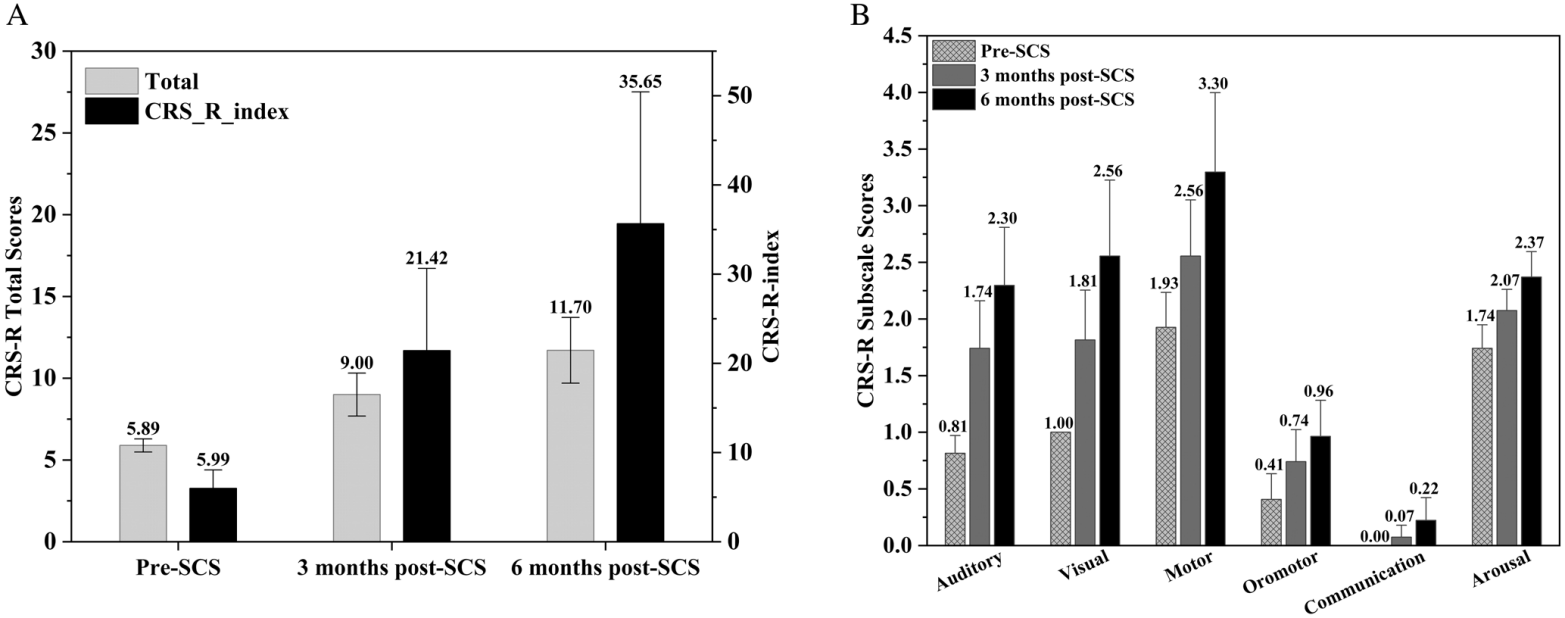
(A) shows the total CRS‐R scores and the CRS‐R index of 27 patients before spinal cord stimulation (SCS), 3 months after surgery, and 6 months after surgery. The left vertical axis represents the total CRS‐R scores, while the right vertical axis represents the CRS‐R index. **indicates that the statistical results show *p* < 0.001 compared to the preoperative values. (B) displays the subscale scores of the CRS‐R for these patients at the aforementioned three time points.

In this study, we compared the CRS‐R scores of patients before surgery and at 3 months and 6 months post‐surgery. The results showed that the total CRS‐R scores at both 3 months and 6 months post‐surgery were significantly improved compared to the pre‐surgery scores, with an average improvement of 3 points at 3 months and over 5 points at 6 months. Notably, the patient with the best awakening effect improved by as much as 15 points at 6 months post‐surgery compared to their pre‐surgery score. Additionally, we also compared the CRS‐R index before surgery and at 3 months and 6 months post‐surgery. The results again showed that the total CRS‐R index at both 3 months and 6 months post‐surgery was significantly improved compared to the pre‐surgery scores, with an average increase of over 15 points at 3 months and over 30 points at 6 months. Furthermore, at 6 months post‐surgery, 9 patients (approximately 69.2%) had a CRS‐R index improvement of more than 2 points, as illustrated in Table 2 and Figure 1.

**TABLE 2:** The CRS‐R scores of each patient before spinal cord stimulation (SCS), 3 months after surgery, and 6 months after surgery, along with the calculated CRS‐R‐index.

Patients	Pre‐SCS		3 months post‐SCS		6 months post‐SCS	
	CRS‐R	CRS‐R‐index	CRS‐R	CRS‐R‐index	CRS‐R	CRS‐R‐index
1	6 (1–1–2‐0‐0‐2)	4.84	6 (1–1–2‐0‐0‐2)	4.84	6 (1–1–2‐0‐0‐2)	4.84
2	5 (1–1–1‐0‐0‐2)	3.80	5 (1–1–1‐0‐0‐2)	3.80	5 (1–1–1‐0‐0‐2)	3.80
3	7 (1–1–2‐1‐0‐2)	5.88	8 (2–1–2‐1‐0‐2)	6.92	8 (2–1–2‐1‐0‐2)	6.92
4	5 (0–1–2‐0‐0‐2)	3.80	9 (2–2–2‐1‐0‐2)	15.26	18 (4–4–6‐1‐0‐3)	82.28
5	7 (1–1–2‐1‐0‐2)	5.88	17 (3–3–5‐2‐1‐3)	66.65	22 (4–5–6‐2‐2‐3)	NA
6	8 (1–1–5‐0‐0‐1)	29.51	8 (1–1–5‐0‐0‐1)	29.51	21 (4–5–6‐2‐1‐3)	100.00
7	6 (1–1–2‐0‐0‐2)	4.84	6 (1–1–2‐0‐0‐2)	4.84	6 (1–1–2‐0‐0‐2)	4.84
8	6 (1–1–2‐1‐0‐1)	5.54	6 (1–1–2‐1‐0‐1)	5.54	6 (1–1–2‐1‐0‐1)	5.54
9	6 (0–1–2‐1‐0‐2)	4.84	7 (1–1–2‐1‐0‐2)	5.88	15 (3–3–5‐1‐0‐3)	57.27
10	7 (1–1–2‐1‐0‐2)	5.88	16 (2–3–5‐2‐1‐3)	58.32	21 (4–5–6‐2‐1‐3)	100.00
11	5 (1–1–2‐0‐0‐1)	4.50	8 (2–2–2‐0‐0‐2)	14.22	10 (2–3–3‐0‐0‐2)	30.89
12	4 (1–1–2‐0‐0‐0)	4.17	7 (2–1–2‐0‐0‐2)	5.88	7 (2–1–2‐0‐0‐2)	5.88
13	5 (1–1–1‐0‐0‐2)	3.80	5 (1–1–1‐0‐0‐2)	3.80	5 (1–1–1‐0‐0‐2)	3.80
14	5 (1–1–1‐0‐0‐2)	3.80	11 (2–3–3‐1‐0‐2)	31.93	16 (3–4–4‐2‐0‐3)	58.32
15	5 (1–1–2‐0‐0‐1)	4.50	17 (4–4–4‐2‐0‐3)	66.65	18 (4–5–4‐2‐0‐3)	74.99
16	5 (0–1–2‐0‐0‐2)	3.80	5 (0–1–2‐0‐0‐2)	3.80	5 (0–1–2‐0‐0‐2)	3.80
17	6 (1–1–2‐0‐0‐2)	4.84	12 (4–3–2‐1‐0‐2)	40.27	18 (4–4–6‐1‐0‐3)	82.28
18	6 (1–1–2‐0‐0‐2)	4.84	7 (1–1–2‐1‐0‐2)	5.88	7 (1–1–2‐1‐0‐2)	5.88
19	7 (1–1–2‐1‐0‐2)	5.88	17 (4–4–5‐1‐0‐3)	73.95	21 (4–5–6‐2‐1‐3)	100.00
20	4 (0–1–1‐0‐0‐2)	2.75	5 (1–1–1‐0‐0‐2)	3.80	6 (1–1–2‐0‐0‐2)	4.84
21	6 (1–1–2‐0‐0‐2)	4.84	10 (2–3–2‐1‐0‐2)	23.60	11 (2–3–3‐1‐0‐2)	31.93
22	7 (1–1–2‐1‐0‐2)	5.88	14 (3–4–4‐1‐0‐2)	56.94	20 (4–5–5‐2‐1‐3)	91.66
23	7 (1–1–2‐1‐0‐2)	5.88	7 (1–1–2‐1‐0‐2)	5.88	7 (1–1–2‐1‐0‐2)	5.88
24	5 (1–1–1‐0‐0‐2)	3.80	6 (1–1–2‐0‐0‐2)	4.84	8 (2–2–2‐0‐0‐2)	14.22
25	4 (0–1–1‐0‐0‐2)	2.75	6 (1–1–2‐0‐0‐2)	4.84	8 (2–1–2‐1‐0‐2)	6.92
26	9 (1–1–3‐2‐0‐2)	15.26	11 (2–2–3‐2‐0‐2)	24.64	13 (2–3–3‐2‐0‐3)	33.31
27	6 (1–1–2‐1‐0‐1)	5.54	7 (1–1–2‐1‐0‐2)	5.88	8 (2–1–2‐1‐0‐2)	6.92

*Note:* The subscale scores in parentheses are listed in the order of Auditory, Visual, Motor, Oromotor, Communication, and Arousal.

### 3.3 Power Spectrum Results

**FIGURE 2:**
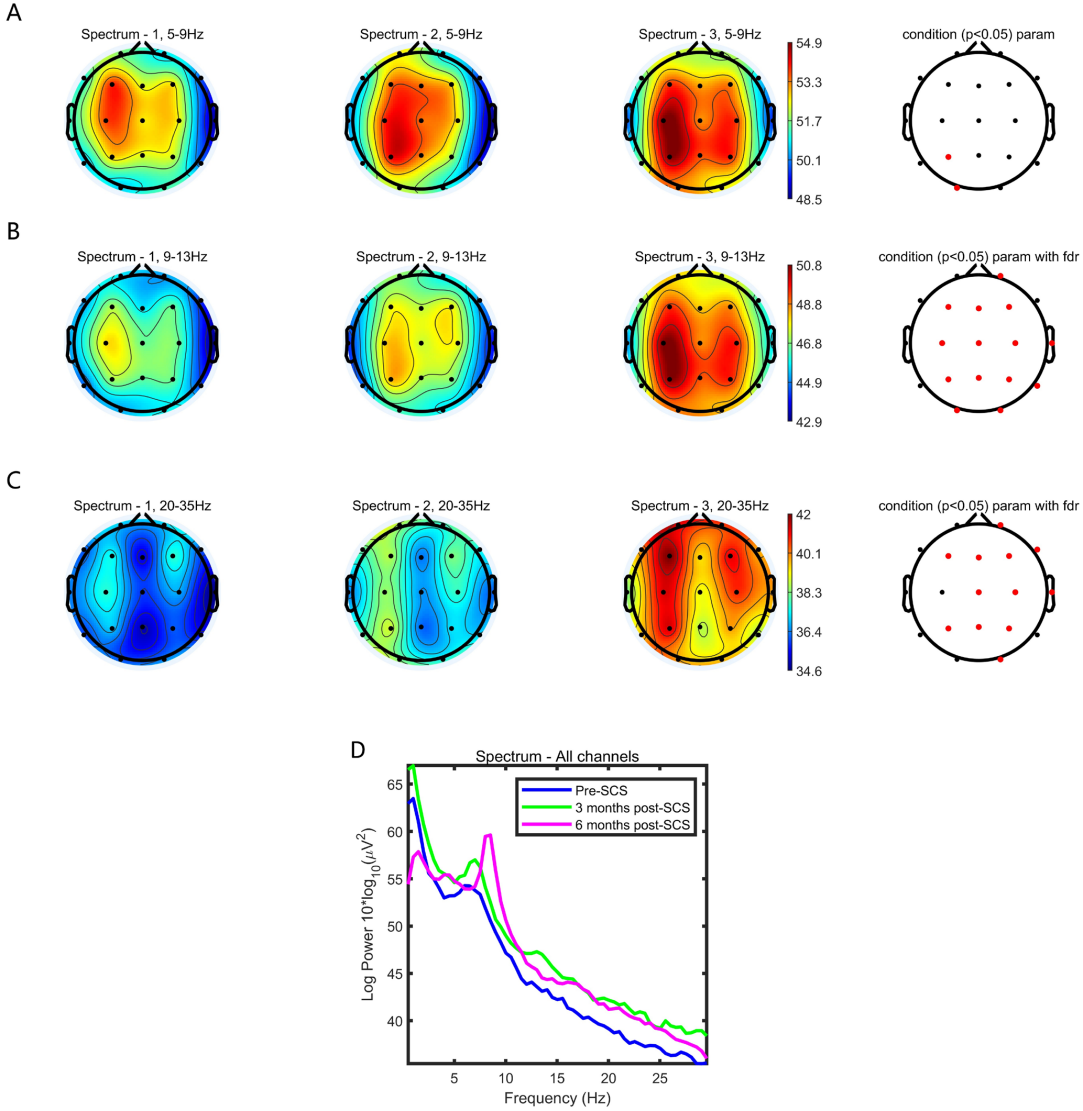
(A) represents the topographical map of the power spectrum for the 5–9 Hz frequency band; (B) represents the topographical map of the power spectrum for the 9–13 Hz frequency band; (C) represents the topographical map of the power spectrum for the 20–35 Hz frequency band. The electrode sites highlighted in red indicate that the power values at those electrodes have statistical significance. The electrode sites with statistical significance in Figures (B) and (C) have survived FDR correction, while those in Figure (A) have not. (D) shows the changes in scalp electroencephalogram power spectral density for one patient before and after surgery, with a transition from Type B before surgery to Type D 6 months post‐surgery.

In this study, we collected resting‐state EEG data from 13 patients who underwent SCS surgery between May 2023 and November 2023, at preoperative, 3 months postoperative, and 6 months postoperative time points. We used the FFT method to obtain the power spectra and performed relevant statistical calculations, applying FDR correction to the statistical results. We found that the power values in the frequency bands of 5–9 Hz, 9–13 Hz, and 20–35 Hz increased compared to preoperative levels, especially at 6 months post‐surgery, where significant improvements were observed. However, the 5–9 Hz band showed statistical significance only at the P3 and O1 electrodes and do not survive FDR correction. In contrast, the power values in the 9–13 Hz and 20–35 Hz bands had statistical significance in the majority of the 19 electrodes (*p* < 0.05) and survive FDR correction. Table 3 presents the assessment results of the “ABCD” model for each patient at preoperative, 3 months postoperative, and 6 months postoperative time points, along with the patients' consciousness states evaluated according to the CRS‐R index. Figure 2 displays the topographical maps of the power spectra for each frequency band at preoperative, 3 months postoperative, and 6 months postoperative time points, D shows the changes in the power spectral density maps of one patient at preoperative, 3 months postoperative, and 6 months postoperative time points.

**TABLE 3:** Results of the “ABCD” model assessment for each patient before the SCS procedure, 3 months after surgery, and 6 months after surgery, along with the corresponding consciousness states.

Patients	Pre‐SCS		3 months post‐SCS		6 months post‐SCS	
	“ABCD” model	Behavioral diagnoses	“ABCD” model	Behavioral diagnoses	“ABCD” model	Behavioral diagnoses
15	B	UWS	B	MCS	C	MCS
16	B	UWS	B	UWS	A	UWS
17	B	UWS	B	MCS	C	MCS
18	B	UWS	B	UWS	C	UWS
19	B	UWS	B	MCS	C	MCS
20	B	UWS	B	UWS	B	UWS
21	B	UWS	B	MCS	B	MCS
22	B	UWS	B	MCS	C	MCS
23	B	UWS	B	UWS	B	UWS
24	B	UWS	B	UWS	B	MCS
25	B	UWS	B	UWS	B	UWS
26	B	MCS	B	MCS	D	MCS
27	B	UWS	B	UWS	C	UWS

*Note:* The consciousness state is determined based on the calculated CRS‐R score, with scores above 8.315 indicating MCS (Minimally Conscious State) and scores below 8.315 indicating UWS (Unresponsive Wakefulness Syndrome) [17].

### 3.4 ERP Results

**FIGURE 3:**
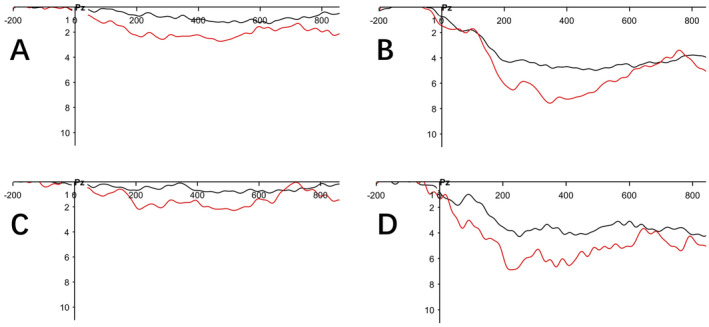
(A) shows the average waveform of P300 at the Pz electrode for 27 patients preoperatively; (B) shows the average waveform of P300 at the Pz electrode for 27 patients 3 months postoperatively; (C) shows the average waveform of P300 at the Pz electrode for 15 patients preoperatively; (D) shows the average waveform of P300 at the Pz electrode for 15 patients 6 months postoperatively. The black line represents the standard stimulus, while the red line represents the deviant stimulus.

In this study, we compared the mean amplitude, peak, and latency of P300 obtained using midline electrodes (Fz, CZ, and Pz) before and after SCS surgery. According to the statistical results, we found that the average amplitude and peak of P300 at the Pz electrode significantly increased compared to preoperative levels, while the latency was significantly shortened. Statistical analysis showed that all findings were statistically significant (*p* < 0.001). Table 4 presents a description of the indicators of P300 at the Pz electrode before surgery and 3 months after surgery under both standard stimulus and deviant stimulus conditions. At 6 months post‐surgery, we reassessed ERP in 15 patients from the aforementioned group, and compared to preoperative levels, the average amplitude and peak still showed significant increases (*p* < 0.05), while the latency remained significantly shortened (*p* < 0.001). Table 5 presents a description of the indicators of P300 at the Pz electrode before surgery and 6 months after surgery under both standard stimulus and deviant stimulus conditions. Figure 3 shows the group average waveforms of ERP at preoperative, 3 months postoperative, and 6 months postoperative time points.

**TABLE 4:** Mean amplitude, peak, and latency of P300 at the Pz electrode before surgery and 3 months after surgery under standard stimulus and deviant stimulus conditions.

	Standard stimulus			Deviant stimulus		
	Mean amplitude	Peak	Latency	Mean amplitude	Peak	Latency
Pre‐SCS	(0.000, 1.954)	(0.000, 2.569)	(440, 548)	(0.832, 3.948)	(2.489, 5.404)	(436, 544)
Post‐SCS	(1.320, 7.659)	(2.196, 9.308)	(314, 378)	(4.215, 10.004)	(6.249, 7.937)	(332, 370)
*p*	< 0.001**	< 0.001**	< 0.001**	< 0.001**	< 0.001**	< 0.001**
*Z*	−3.492	−3.594	−4.517	−3.580	−3.556	−4.469

** *p* < 0.001.

**TABLE 5:** Mean amplitude, peak, and latency of P300 at the Pz electrode before surgery and 6 months after surgery under standard stimulus and deviant stimulus conditions.

	Standard stimulus			Deviant stimulus		
	Mean amplitude	Peak	Latency	Mean amplitude	Peak	Latency
Pre‐SCS	(0.000, 0.954)	(0.000, 1.811)	491.47 ± 18.88	(0.789, 2.766)	(1.908, 4.735)	488.53 ± 14.43
Post‐SCS	(0.536, 7.132)	(1.951, 7.748)	375.73 ± 4.50	(2.675, 9.120)	(3.313, 9.951)	373.20 ± 4.16
*p*	0.017*	0.02*	< 0.001**	0.011*	0.041*	< 0.001**
Z/F	−2.385	−1.161	0.343	−1.817	−2.045	0.803

* *p* < 0.05.

** *p* < 0.001.

## 4 Discussion

Many studies have reported the clinical effects of spinal cord stimulation (SCS) surgery on patients with Disorders of Consciousness (DOC) [37, 38]. However, most conclusions regarding improvements in DOC patients are based solely on changes in CRS‐R scores, which can be subjectively influenced by clinicians. Some studies have also explored objective assessment methods, such as TMS‐EEG [39] and permutation entropy (PeEn) [3] of neural activities, to investigate the improvements in DOC patients after SCS. Our research aims to find clinically applicable objective methods to detect whether the consciousness state of patients has improved following SCS surgery. We also hope that these assessment methods can play a role in screening patients during preoperative evaluations to improve the success rate of the surgery.

In this study, we assessed whether the SCS surgery achieved its purpose of awakening by evaluating improvements in the CRS‐R index (an increase of ≥ 2 points). Among the 27 pDOC patients who underwent SCS surgery, 16 patients showed an increase in the CRS‐R index of more than 2 points, resulting in an effective awakening rate of 59.2%. In a meta‐analysis article involving 27 relevant studies, it was mentioned that approximately 26% of patients showed clinical improvement, and among them, only 17% could be considered as having achieved full consciousness recovery [40]. Additionally, an international multicenter study reported that about 30% of patients with disorders of consciousness exhibited clinical improvement 6 months after the onset of their condition [41]. It can be seen that SCS surgery can effectively improve the clinical improvement rate in patients with disorders of consciousness. We defined these 16 patients as the responder group, while the remaining 11 patients constituted the non‐responder group. Based on preoperative demographics, the male‐to‐female ratio in the responder group was 5:3, whereas in the non‐responder group it was 6:5. The average age of the responder group was 51.9 years (ranging from 23 to 68 years), with an average duration of illness of 5.4 months (ranging from 2 to 13 months). In comparison, the non‐responder group had an average age of 46.1 years (ranging from 22 to 65 years) and an average illness duration of 4.5 months (ranging from 2 to 12 months). In the responder group, there were nine cases of intracerebral hemorrhage and seven cases of traumatic brain injury. In the non‐responder group, there were six cases of intracerebral hemorrhage, three cases of traumatic brain injury, and additionally, two cases of ischemic–hypoxic encephalopathy. From the above data, it can be seen that the patients' age, gender, and average duration of illness at the time of surgery do not significantly influence the effective awakening rate of SCS surgery for pDOC patients. However, considering the etiology, the effective rates were 60% (9/15) for patients with intracerebral hemorrhage and 70% (7/10) for patients with traumatic brain injury, while both cases of ischemic–hypoxic encephalopathy were in the non‐responder group. This indicates that the underlying cause of consciousness impairment has a significant impact on the effectiveness of SCS surgery for awakening.

An educated inspection of basic EEG features in pDoC can provide relevant information about the functional integrity of thalamocortical networks [22]. Our current understanding of the mechanisms of brain oscillations [42, 43] suggests that at least some of the EEG patterns observed in DoC may reflect disconnection (or deafferentation) within and among cortical and subcortical structures [22, 44]. Extensive macroscopic structural disconnections of cortical circuits are known to produce an EEG pattern dominated by oscillations in the delta rang [45]. The proposed ‘ABCD’ model [22, 29] shows the correlation of coarsely divided levels of cerebral deafferentation in severe brain injuries and the EEG power spectrum changes predicted to occur based on functional changes within neocortical and thalamic neurons as a result of the deafferentation patterns. A range of pathologic processes likely interact with individual structural injury patterns producing a mix of the signals associated with these EEG power spectral categories: A (reflecting structurally or functionally isolated cortex), B (sharply gated intrinsically generated cortical oscillations), C (patterns driven by thalamic bursting in wakeful states generating coupled theta/beta‐gamma rhythms), and D (normal background activity). According to the literature, Type C shows oscillations in the 20–35 Hz frequency range compared to Type B, while Type D exhibits oscillations in the 9–13 Hz frequency range compared to Type C [29]. Additionally, Types B and C can correspond to the behaviorally diagnosed minimally conscious state (MCS). Healthy intact neurons in the neocortex show as modeled as “D‐type” spectra [20].

In this study, the “ABCD” model was introduced in May 2023 as a tool for clinical preoperative assessment, using Type B or C as surgical indications. Subsequently, 13 patients were selected to undergo spinal cord stimulation (SCS) surgery. Based on postoperative follow‐up using the CRS‐R index, the effective awakening rate after implementing this model was 69.2% (9/13), compared to an effective rate of 50.0% (7/14) before its implementation. The introduction of this model effectively improved the success rate of SCS surgery. Additionally, we compared the changes in scalp EEG power spectrum before and after surgery, finding that the power values in the frequency ranges of 9–13 Hz and 20–35 Hz increased postoperatively, indicating that patients were transitioning from Type B to Types C and D. This suggests a gradual improvement in the patients' state of consciousness, which aligns with the results evaluated by the CRS‐R score and the CRS‐R index. Furthermore, we observed that changes in the power spectrum may occur earlier than changes reflected in behavioral scales. ERPs, mainly the P300 component, have been widely used to detect the electrophysiological correlates of cognitive capabilities, potentially reflecting residual levels of awareness in patients with DOCs [46, 47]. According to one of the most important meta‐analyses of ERPs in comatose patients [48], MMN and P300 should be considered as significant positive but not negative early predictors in comatose patients.

In our study, we used “passive” paradigms that are independent of the patient's collaboration and have been shown to have significantly fewer limitations than “active” paradigms, for example, misunderstanding of task instructions by the patients. The ultimate goal when establishing an ERP task for patients with severe brain injury is not only to elicit cognitively mediated responses but also to not exceed the cognitive capacity of the patients. We believe that actively listening to pitch or counting target stimuli requires higher cognitive capacities compared to just listening. In addition, active ERP tasks require the patients not only to stay awake during the recording but also to understand the commands, be able to hold perceptual representations in their working memory, and complete the task [49].

The P300 amplitude depends not only on the stimulus saliency but also on the participant's attentiveness [50, 51]. Previous studies have reported that there is a variation in the P300 amplitude according to the amount of focal attention on discriminate stimuli. It can be speculated that the observed SCS‐related consciousness improvements (as assessed by changes in the CRS‐R score) are potentially related to improvements in attention resource allocation (as reflected by the P300 mean amplitude and peak). In our study, we also found that the P300 latency at the Pz electrode significantly decreased compared to preoperative levels, indicating an improvement in the patients' ability to process information requiring higher attention after surgery [52].

Although the results are exciting, some questions remain to be addressed in future studies. First, the main limitations of this study are the small sample size and lack of long‐term follow‐up. Second, due to the small sample size, our power spectrum study lacked further subgrouping of patients with different etiologies of brain tissue damage, which would help exclude the effects of damage in various brain regions on the EEG power spectrum. In future studies, the sample size should be increased, and patients with different etiologies and areas of brain injury should be further grouped to mitigate the influence of etiology on the power spectrum analysis. Thirdly, the EEG data collected postoperatively in this study were obtained while the SCS system was turned off, which somewhat diminished the observed effects of SCS on improving consciousness states. In future studies, we should collect EEG data from patients in both the on and off states of the device for comparison. Of course, this requires addressing how to successfully extract valid EEG signals from the artifacts caused by SCS electrical stimulation. This study aims to explore the relevant changes in behavioral scale data and scalp EEG in patients with pDOC after SCS surgery. Based on clinical observations, we have not found significant differences in postoperative efficacy between patients of different sexes. Additionally, all patients in this study are individuals with impaired consciousness who are unable to express their gender identity. Therefore, this study does not address the effects related to sex and gender identity.

## 5 Conclusion

Our study shows that SCS can effectively improve the consciousness states of patients with DOC. After surgery, there were positive changes in the EEG power spectrum of the patients, transitioning from Type “B” to better Types “C” and “D.” The average amplitude, peak, and latency of P300 also demonstrated significant improvements postoperatively. We believe that the “ABCD” model and ERP assessment applied during the preoperative evaluation can effectively enhance the success rate of SCS surgery in promoting awakening.

## Consent

All the authors have approved the final version of the manuscript for publication.

## Conflicts of Interest

The authors declare no conflicts of interest.

## Supporting information


Data S1.

## Data Availability

The data that support the findings of this study are available on request from the corresponding author. The data are not publicly available due to privacy or ethical restrictions.

## References

[1] A. Comanducci , M. Boly , J. Claassen , et al., “Clinical and Advanced Neurophysiology in the Prognostic and Diagnostic Evaluation of Disorders of Consciousness: Review of an IFCN‐Endorsed Expert Group,” Clinical Neurophysiology: Official Journal of the International Federation of Clinical Neurophysiology 131, no. 11 (2020): 2736–2765, 10.1016/j.clinph.2020.07.015. 32917521

[2] S. Laureys , G. G. Celesia , F. Cohadon , et al., “Unresponsive Wakefulness Syndrome: A New Name for the Vegetative State or Apallic Syndrome,” BMC Medicine 8 (2010): 8. 21040571 10.1186/1741-7015-8-68 PMC2987895

[3] Y. Wang , Y. Bai , X. Xia , Y. Yang , J. He , and X. Li , “Spinal Cord Stimulation Modulates Complexity of Neural Activities in Patients With Disorders of Consciousness,” International Journal of Neuroscience 130, no. 7 (2020): 662–670, 10.1080/00207454.2019.1702543. 31847650

[4] B. Kotchoubey , D. Vogel , S. Lang , and F. Müller , “What Kind of Consciousness Is Minimal?,” Brain Injury 28, no. 9 (2014): 1156–1163, 10.3109/02699052.2014.920523. 25099020

[5] A. Candelieri , M. D. Cortese , G. Dolce , F. Riganello , and W. G. Sannita , “Visual Pursuit: Within‐Day Variability in the Severe Disorder of Consciousness,” Journal of Neurotrauma 28, no. 10 (2011): 2013–2017, 10.1089/neu.2011.1885. 21770758

[6] R. Formisano , M. D'Ippolito , and S. Catani , “Functional Locked‐In Syndrome as Recovery Phase of Vegetative State,” Brain Injury 27 (2013): 27(11), 10.3109/02699052.2013.809555. 23927719

[7] J. Luauté , D. Maucort‐Boulch , L. Tell , et al., “Long‐Term Outcomes of Chronic Minimally Conscious and Vegetative States,” Neurology 75, no. 3 (2010): 246–252, 10.1212/WNL.0b013e3181e8e8df. 20554940

[8] F. M. Hammond , J. T. Giacino , R. Nakase Richardson , et al., “Disorders of Consciousness due to Traumatic Brain Injury: Functional Status Ten Years Post‐Injury,” Journal of Neurotrauma 36, no. 7 (2019): 1136–1146, 10.1089/neu.2018.5954. 30226400

[9] M. A. Bruno , A. Vanhaudenhuyse , A. Thibaut , G. Moonen , and S. Laureys , “From Unresponsive Wakefulness to Minimally Conscious PLUS and Functional Locked‐In Syndromes: Recent Advances in Our Understanding of Disorders of Consciousness,” Journal of Neurology 258, no. 7 (2011): 1373–1384, 10.1007/s00415-011-6114-x. 21674197

[10] J. T. Giacino , S. Ashwal , N. Childs , et al., “The Minimally Conscious State: Definition and Diagnostic Criteria,” Neurology 58, no. 3 (2002): 349–353, 10.1212/wnl.58.3.349. 11839831

[11] A. Thibaut , N. Schiff , J. Giacino , S. Laureys , and O. Gosseries , “Therapeutic Interventions in Patients With Prolonged Disorders of Consciousness,” Lancet Neurology 18, no. 6 (2019): 600–614, 10.1016/S1474-4422(19)30031-6. 31003899

[12] T. Yamamoto , Y. Katayama , T. Obuchi , K. Kobayashi , H. Oshima , and C. Fukaya , “Spinal Cord Stimulation for Treatment of Patients in the Minimally Conscious State,” Neurologia Medico‐Chirurgica 52, no. 7 (2012): 475–481, 10.2176/nmc.52.475. 22850495

[13] G. M. Della Pepa , C. Fukaya , G. La Rocca , J. Zhong , and M. Visocchi , “Neuromodulation of Vegetative State Through Spinal Cord Stimulation: Where Are We Now and Where Are We Going?,” Stereotactic and Functional Neurosurgery 91, no. 5 (2013): 275–287, 10.1159/000348271. 23797266

[14] M. Visocchi , G. M. Della Pepa , G. Esposito , et al., “Spinal Cord Stimulation and Cerebral Hemodynamics: Updated Mechanism and Therapeutic Implications,” Stereotactic and Functional Neurosurgery 89, no. 5 (2011): 263–274, 10.1159/000329357. 21860253

[15] J. T. Giacino , K. Kalmar , and J. Whyte , “The JFK Coma Recovery Scale‐Revised: Measurement Characteristics and Diagnostic Utility,” Archives of Physical Medicine and Rehabilitation 85, no. 12 (2004): 2020–2029, 10.1016/j.apmr.2004.02.033. 15605342

[16] Y. Zhang , J. Wang , C. Schnakers , et al., “Validation of the Chinese Version of the Coma Recovery Scale‐Revised (CRS‐R),” Brain Injury 33, no. 4 (2019): 529–533, 10.1080/02699052.2019.1566832. 30663434

[17] J. Annen , M. M. Filippini , E. Bonin , et al., “Diagnostic Accuracy of the CRS‐R Index in Patients With Disorders of Consciousness,” Brain Injury 33, no. 11 (2019): 1409–1412, 10.1080/02699052.2019.1644376. 31319707

[18] D. Morlet and C. Fischer , “MMN and Novelty P3 in Coma and Other Altered States of Consciousness: A Review,” Brain Topography 27 (2014): 467–479, 10.1007/s10548-013-0335-5. 24281786 PMC5034015

[19] R. Li , W. Q. Song , J. B. Du , S. Huo , and G. X. Shan , “Connecting the P300 to the Diagnosis and Prognosis of Unconscious Patients,” Neural Regeneration Research 10, no. 3 (2015): 473–480, 10.4103/1673-5374.153699. 25878599 PMC4396113

[20] N. D. Schiff , “Mesocircuit Mechanisms in the Diagnosis and Treatment of Disorders of Consciousness,” Presse Medicale (Paris, France: 1983) 52, no. 2 (2023): 104161, 10.1016/j.lpm.2022.104161. 36563999

[21] N. D. Schiff , “Recovery of Consciousness After Brain Injury: A Mesocircuit Hypothesis,” Trends in Neurosciences 33, no. 1 (2010): 1–9, 10.1016/j.tins.2009.11.002. 19954851 PMC2931585

[22] N. D. Schiff , “Mesocircuit Mechanisms Underlying Recovery of Consciousness Following Severe Brain Injuries: Model and Predictions,” in Brain Function and Responsiveness in Disorders of Consciousness, eds. M. M. Monti and W. G. Sannita (Cham: Springer International Publishing, 2016), 195–204, 10.1007/978-3-319-21425-2_15.

[23] S. W. Hughes and V. Crunelli , “Thalamic Mechanisms of EEG Alpha Rhythms and Their Pathological Implications,” Neuroscientist: A Review Journal Bringing Neurobiology, Neurology and Psychiatry 11, no. 4 (2005): 357–372, 10.1177/1073858405277450. 16061522

[24] L. Ka , L. Cl , S. Sm , et al., “Thalamic Metabolic Rate Predicts EEG Alpha Power in Healthy Control Subjects but Not in Depressed Patients,” Biological Psychiatry 45, no. 8 (1999): 943–952, 10.1016/s0006-3223(98)00350-3. 10386175

[25] Z. Liu , J. A. de Zwart , B. Yao , P. van Gelderen , L. W. Kuo , and J. H. Duyn , “Finding Thalamic BOLD Correlates to Posterior Alpha EEG,” NeuroImage 63, no. 3 (2012): 1060–1069, 10.1016/j.neuroimage.2012.08.025. 22986355 PMC3472152

[26] J. Sarnthein and D. Jeanmonod , “High Thalamocortical Theta Coherence in Patients With Parkinson's Disease,” Journal of Neuroscience: The Official Journal of the Society for Neuroscience 27 (2007): 124–131, 10.1523/JNEUROSCI.2411-06.2007. 17202479 PMC6672280

[27] J. Sarnthein , A. Morel , A. von Stein , and D. Jeanmonod , “Thalamocortical Theta Coherence in Neurological Patients at Rest and During a Working Memory Task,” International Journal of Psychophysiology : Official Journal of the International Organization of Psychophysiology 57, no. 2 (2005): 87–96, 10.1016/j.ijpsycho.2005.03.015. 15982767

[28] M. Schreckenberger , C. Lange‐Asschenfeldt , M. Lochmann , et al., “The Thalamus as the Generator and Modulator of EEG Alpha Rhythm: A Combined PET/EEG Study With Lorazepam Challenge in Humans,” NeuroImage 22, no. 2 (2004): 637–644, 10.1016/j.neuroimage.2004.01.047. 15193592

[29] B. L. Edlow , J. Claassen , N. D. Schiff , and D. M. Greer , “Recovery From Disorders of Consciousness: Mechanisms, Prognosis and Emerging Therapies,” Nature Reviews Neurology 17, no. 3 (2021): 135–156, 10.1038/s41582-020-00428-x. 33318675 PMC7734616

[30] W. St , C. Mm , G. Am , et al., “Common Resting Brain Dynamics Indicate a Possible Mechanism Underlying Zolpidem Response in Severe Brain Injury,” eLife 2 (2013): 2, 10.7554/eLife.01157. PMC3833342 24252875

[31] P. B. Forgacs , H. P. Frey , A. Velazquez , et al., “Dynamic Regimes of Neocortical Activity Linked to Corticothalamic Integrity Correlate With Outcomes in Acute Anoxic Brain Injury After Cardiac Arrest,” Annals of Clinical and Translational Neurology 4, no. 2 (2017): 119–129, 10.1002/acn3.385. 28168211 PMC5288467

[32] J. Claassen , A. Velazquez , E. Meyers , et al., “Bedside Quantitative Electroencephalography Improves Assessment of Consciousness in Comatose Subarachnoid Hemorrhage Patients,” Annals of Neurology 80, no. 4 (2016): 541–553, 10.1002/ana.24752. 27472071 PMC5042849

[33] J. D. Drover and N. D. Schiff , “A Method for Decomposing Multivariate Time Series Into a Causal Hierarchy Within Specific Frequency Bands,” Journal of Computational Neuroscience 45, no. 2 (2018): 59–82, 10.1007/s10827-018-0691-y. 30062615

[34] S. A. Shah , Y. Goldin , M. M. Conte , et al., “Executive Attention Deficits After Traumatic Brain Injury Reflect Impaired Recruitment of Resources,” NeuroImage Clinical 14 (2017): 14–241, 10.1016/j.nicl.2017.01.010. PMC5288490 28180082

[35] S. A. Shah , M. Mohamadpour , G. Askin , et al., “Focal Electroencephalographic Changes Index Post‐Traumatic Confusion and Outcome,” Journal of Neurotrauma 34, no. 19 (2017): 2691–2699, 10.1089/neu.2016.4911. 28462682

[36] R. van Dinteren , M. Arns , M. L. Jongsma , and R. P. Kessels , “P300 Development Across the Lifespan: A Systematic Review and Meta‐Analysis,” PLoS One 9, no. 2 (2014): e87347, 10.1371/journal.pone.0087347. 24551055 PMC3923761

[37] G. S. Piedade , B. Assumpcao de Monaco , J. D. Guest , and J. G. Cordeiro , “Review of Spinal Cord Stimulation for Disorders of Consciousness,” Current Opinion in Neurology 36, no. 6 (2023): 507–515, 10.1097/WCO.0000000000001222. 37889524

[38] Y. Wu , Y. Y. Xu , H. Deng , et al., “Spinal Cord Stimulation and Deep Brain Stimulation for Disorders of Consciousness: A Systematic Review and Individual Patient Data Analysis of 608 Cases,” Neurosurgical Review 46, no. 1 (2023): 200, 10.1007/s10143-023-02105-1. 37578633

[39] Y. Wang , Y. Dang , Y. Bai , X. Xia , and X. Li , “Evaluating the Effect of Spinal Cord Stimulation on Patient With Disorders of Consciousness: A TMS‐EEG Study,” Computers in Biology and Medicine 166 (2023): 107547, 10.1016/j.compbiomed.2023.107547. 37806053

[40] A. Magliacano , F. de Bellis , F. Panico , et al., “Long‐Term Clinical Evolution of Patients With Prolonged Disorders of Consciousness due to Severe Anoxic Brain Injury: A Meta‐Analytic Study,” European Journal of Neurology 30, no. 12 (2023): 3913–3927, 10.1111/ene.15899. 37246500

[41] A. Estraneo , S. Fiorenza , A. Magliacano , et al., “Multicenter Prospective Study on Predictors of Short‐Term Outcome in Disorders of Consciousness,” Neurology 95, no. 11 (2020): e1488–e1499, 10.1212/WNL.0000000000010254. 32661102 PMC7713739

[42] M. Steriade , “Grouping of Brain Rhythms in Corticothalamic Systems,” Neuroscience 137, no. 4 (2006): 1087–1106, 10.1016/j.neuroscience.2005.10.029. 16343791

[43] M. V. Sanchez‐Vives , M. Massimini , and M. Mattia , “Shaping the Default Activity Pattern of the Cortical Network,” Neuron 94, no. 5 (2017): 993–1001, 10.1016/j.neuron.2017.05.015. 28595056

[44] N. D. Schiff , T. Nauvel , and J. D. Victor , “Large‐Scale Brain Dynamics in Disorders of Consciousness,” Current Opinion in Neurobiology 25 (2014): 7–14, 10.1016/j.conb.2013.10.007. 24709594 PMC3980494

[45] P. Gloor , G. Ball , and N. Schaul , “Brain Lesions That Produce Delta Waves in the EEG,” Neurology 27 (1977): 27(4)–333, 10.1212/wnl.27.4.326. 557774

[46] R. G. L. Real , S. Veser , H. Erlbeck , et al., “Information Processing in Patients in Vegetative and Minimally Conscious States,” Clinical Neurophysiology: Official Journal of the International Federation of Clinical Neurophysiology 127, no. 2 (2016): 1395–1402, 10.1016/j.clinph.2015.07.020. 26315366

[47] A. Ragazzoni , M. Cincotta , F. Giovannelli , et al., “Clinical Neurophysiology of Prolonged Disorders of Consciousness: From Diagnostic Stimulation to Therapeutic Neuromodulation,” Clinical Neurophysiology: Official Journal of the International Federation of Clinical Neurophysiology 128, no. 9 (2017): 1629–1646, 10.1016/j.clinph.2017.06.037. 28728060

[48] J. Daltrozzo , N. Wioland , V. Mutschler , and B. Kotchoubey , “Predicting Coma and Other Low Responsive Patients Outcome Using Event‐Related Brain Potentials: A Meta‐Analysis,” Clinical Neurophysiology: Official Journal of the International Federation of Clinical Neurophysiology 118, no. 3 (2007): 606–614, 10.1016/j.clinph.2006.11.019. 17208048

[49] S. L. Hauger , C. Schnakers , S. Andersson , et al., “Neurophysiological Indicators of Residual Cognitive Capacity in the Minimally Conscious State,” Behavioural Neurology 2015 (2015): 1–12, 10.1155/2015/145913. PMC4609423 26504351

[50] W. S. Pritchard , “Psychophysiology of P300,” Psychological Bulletin 89, no. 3 (1981): 506–540. 7255627

[51] R. Johnson, Jr. , “A Triarchic Model of P300 Amplitude,” Psychophysiology 23, no. 4 (1986): 367–384, 10.1111/j.1469-8986.1986.tb00649.x. 3774922

[52] M. Cavinato , C. Volpato , S. Silvoni , M. Sacchetto , A. Merico , and F. Piccione , “Event‐Related Brain Potential Modulation in Patients With Severe Brain Damage,” Clinical Neurophysiology: Official Journal of the International Federation of Clinical Neurophysiology 122, no. 4 (2011): 719–724, 10.1016/j.clinph.2010.08.024. 21051281

