# Supplementary material for: Spinal Cord Stimulation for Prolonged Disorders of Consciousness: A Study on Scalp Electroencephalography
Source: CNS Neurosci Ther. 2024 Dec 30;30(12):e70180. doi: 10.1111/cns.70180 (PMC11683476; doi:10.1111/cns.70180)
Supplement: Supplementary file 1 — Data S1. [file CNS-30-e70180-s001.pdf]

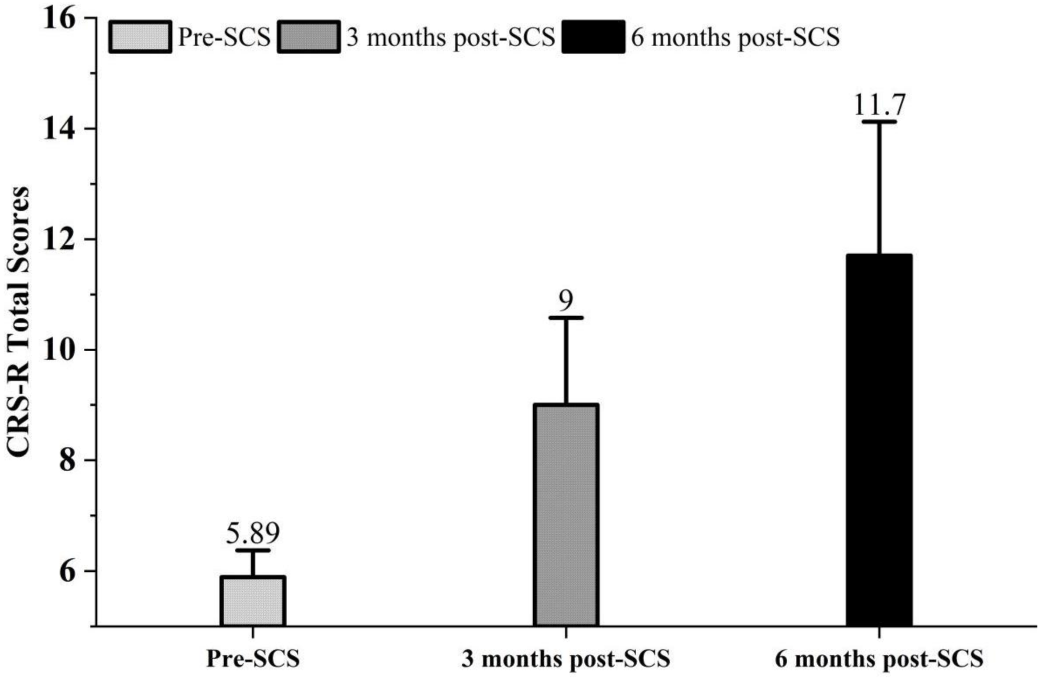

Full unedited gelblot for Figure 1_A

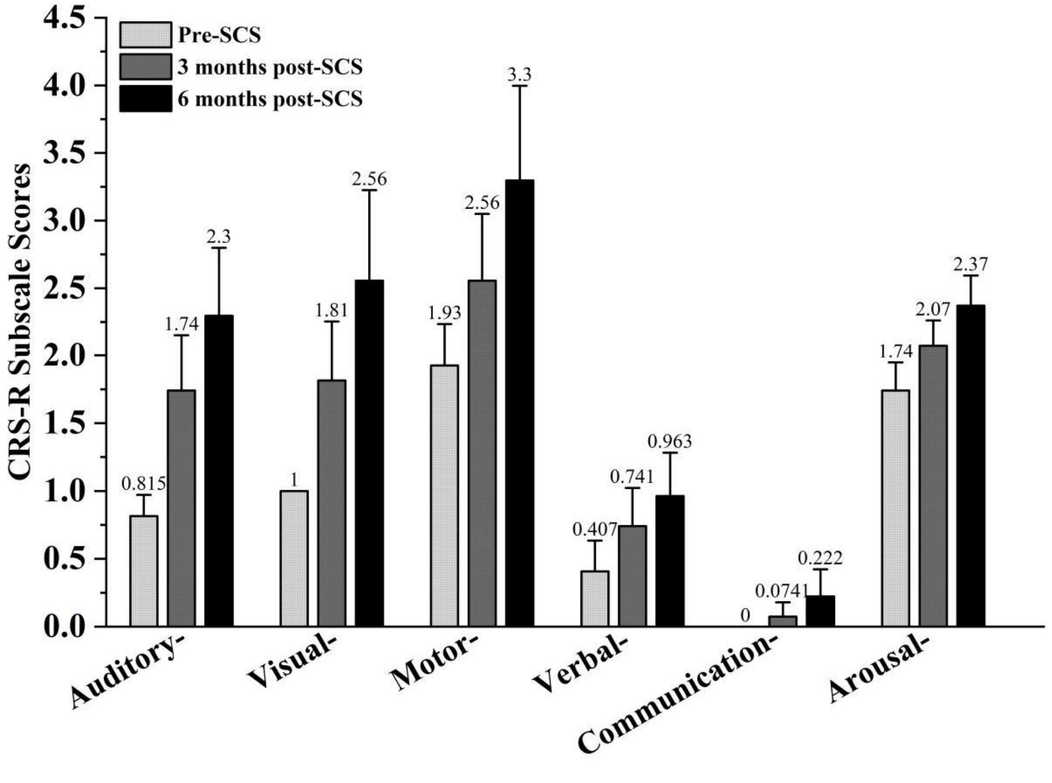

Full unedited gelblot for Figure 1_B

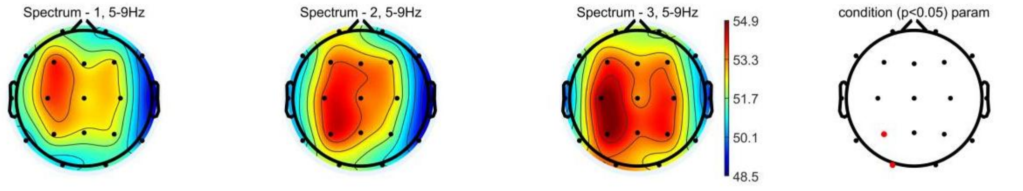

Full unedited gelblot for Figure 2_A

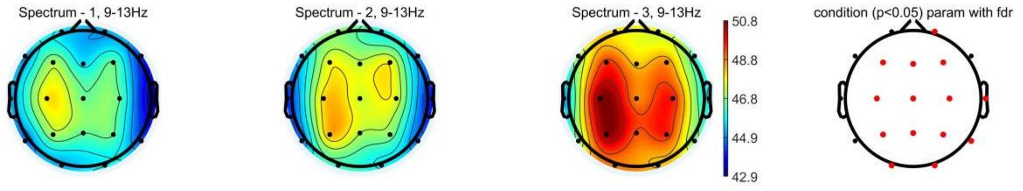

Full unedited gelblot for Figure 2_B

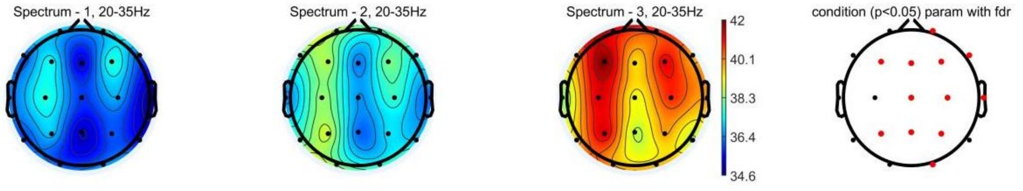

Full unedited gelblot for Figure 2_C

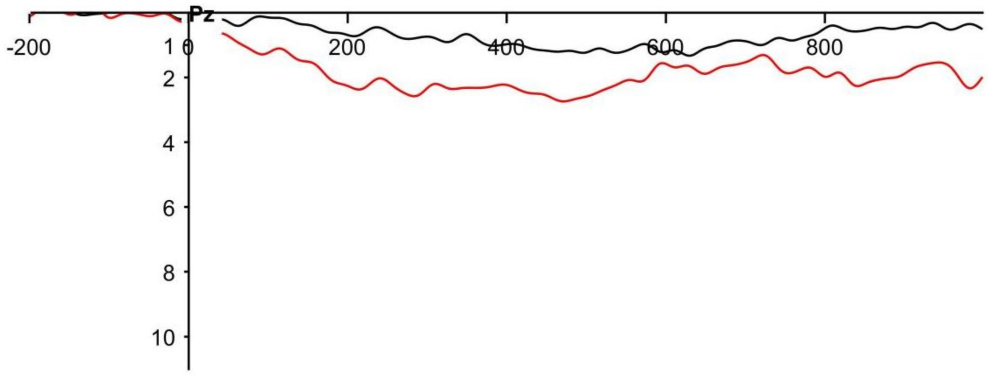

Fll unedited gelblot for Figure 3_A

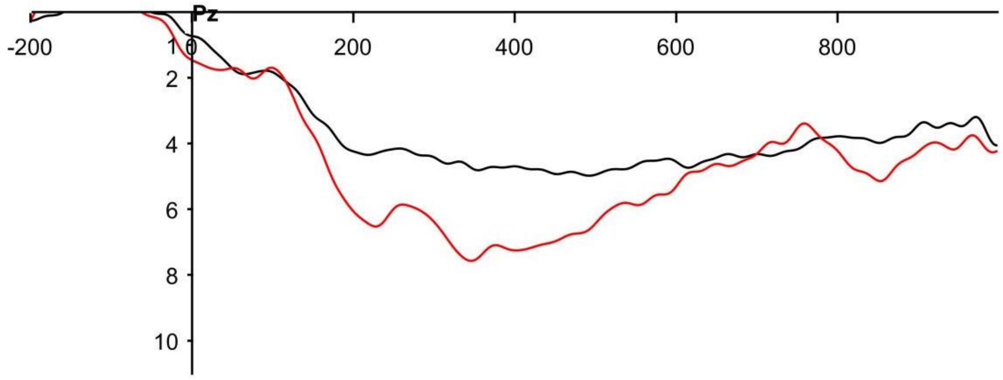

Full unedited gelblot for Figure 3_B

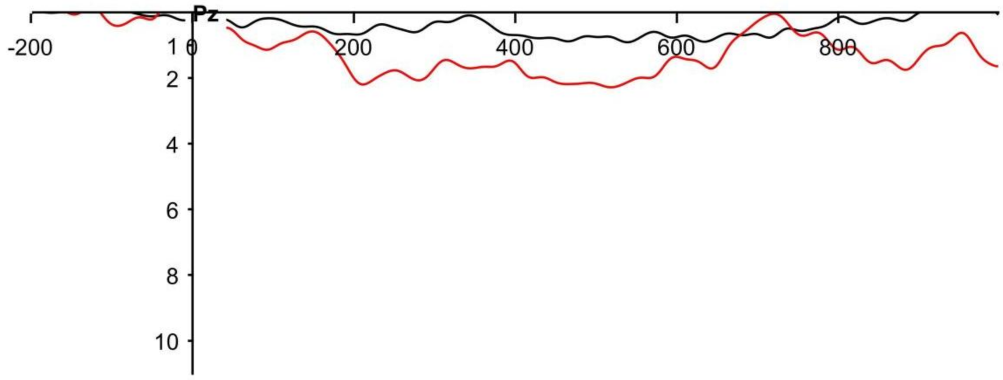

Full unedited gelblot for Figure 3_C

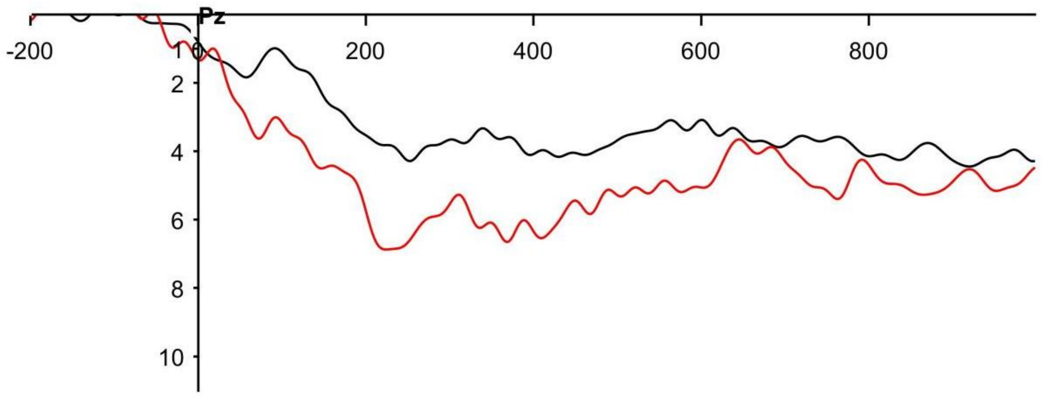

Full

unedited gelblot for Figure 3_D
